# How Time Pressure Amplifies Framing Effects in Risky Decision-Making: The Role of Attentional Allocation and Information Presentation

**DOI:** 10.3390/bs16040548

**Published:** 2026-04-06

**Authors:** Zhun Gong, Haowen Wang, Xiaofei Ma, Yun Lv

**Affiliations:** 1 Department of Psychology, School of Education Science, Qingdao University, Qingdao 266075, China; gongzhun2001@qdu.edu.cn (Z.G.); wanghaowen1@qdu.edu.cn (H.W.); 2 Institute for Educational Measurement and Evaluation, Qingdao University, Qingdao 266075, China; 3 Faculty of Psychology, Tianjin Normal University, Tianjin 300387, China; 2400340002@stu.tjnu.edu.cn

**Keywords:** time pressure, framing effect, risky decision-making, eye-tracking, presentation order, spatial position

## Abstract

Decision-making under time pressure has been associated with reduced deliberation and increased sensitivity to contextual cues such as framing. This study investigates how time pressure reshapes information processing in risky decision-making and which types of information receive greater attention when cognitive resources are constrained. Two experiments examined the combined effects of time pressure, spatial position, and presentation order on framing effects, integrating behavioral risk-choice measures with gaze-based indices of attention allocation. The results show that time pressure significantly reduces fixation counts and fixation durations, suggesting more restricted information search. Moreover, time pressure enhances frame-consistent risk preferences, with contextual presentation factors further shaping decision outcomes. Specifically, under time pressure and loss framing, stronger risk seeking emerged when the certain option was presented second. Overall, these findings suggest that time pressure not only amplifies framing effects in risky decision-making but also is associated with changes in attentional allocation patterns and increased reliance on contextual cues underlying framed choices. This study highlights how the temporal and spatial characteristics of information presentation shape decision processes under temporal constraint and provides theoretical and practical implications for decision-making under pressure.

## 1. Introduction

As uncertainty in real-world decision environments continues to increase, risky decision-making has become a central topic in behavioral decision research, spanning domains such as public policy, financial investment, medical choice, and everyday consumption. Across these contexts, individuals continuously weigh potential losses against gains (Ding et al., 2025). A substantial body of research demonstrates that decision-makers in real settings rarely engage in the comprehensive and dispassionate expected value calculations assumed by classical rational models (Simon, 1978). Instead, under constraints of limited information and time pressure, they rely more heavily on intuitive and experience-based judgment strategies. The framing effect represents one of the most robust and representative manifestations of such bounded rationality (Kühberger, 2023). Specifically, framing effects refer to the systematic tendency for individuals to prefer the certain option when outcomes are framed as gains but to prefer the risky option when the same outcomes are framed as losses, even when the underlying outcomes are logically equivalent (Tversky & Kahneman, 1981). This preference reversal demonstrates that risky decision-making is shaped not only by objective probabilities and outcome attributes but also by the way information is presented and by individuals’ cognitive processing modes.

At the same time, risky decisions in real life rarely occur under calm and undisturbed conditions; instead, they are often made under clear time constraints. Whether it is an emergency physician making a split-second judgment in the operating room, a trader rapidly confirming a transaction in a volatile stock market, or a consumer making a hurried choice before a limited-time discount expires, time pressure profoundly influences how people evaluate risks and rewards. As a key feature of naturalistic decision contexts, time pressure refers to a state in which individuals must make decisions within a limited time frame. Such constraints not only induce anxiety and increase cognitive load but also systematically alter information-processing strategies (Zhou et al., 2024). This raises a critical question: how do “hasty decisions” made under limited time resources affect the manifestation of the framing effect? This issue has become a focal concern in recent behavioral decision research and represents a key entry point for understanding real-world decision behavior (Belli et al., 2023; Guo et al., 2017). Examining this question in depth not only helps clarify the mechanisms underlying risk judgment in natural settings but also contributes to improving the modeling and interpretation of decision patterns in large-scale social data.

Early research primarily explained the framing effect through Prospect Theory, which posits that individuals assign asymmetric subjective weights to gains and losses and are more sensitive to losses than to equivalent gains (Tversky & Kahneman, 1981). More recent work, however, suggests that framing effects are more closely tied to modes of information processing (McElroy, 2024). For decades, dominant dual-system models have provided a theoretical account of this link, proposing that risky decision-making relies on two cognitive systems: System 1, which is fast, intuitive, and affective, and System 2, which is slow, deliberative, and resource-dependent (De Neys & Pennycook, 2019; Kahneman & Frederick, 2007; Grayot, 2019). Critically, System 1 primarily relies on past experience and heuristic processing and lacks the capacity to recognize the normative equivalence of options presented under different frames (Tversky & Kahneman, 1981; Guo et al., 2017). As a result, it is highly sensitive to the surface framing of outcomes and generates rapid affective responses to descriptions of gains and losses—for example, favoring certain options in gain frames while exhibiting greater risk seeking in loss frames. In contrast, System 2 depends on cognitive effort and working memory and, when sufficient time and resources are available, supports the identification of equivalence across frames, the computation of expected values, and the monitoring and correction of intuitive biases generated by System 1 (Kahneman & Frederick, 2007). From this competitive perspective, when external conditions constrain the operation of System 2—such as under time pressure or increased cognitive load—its analytic and corrective functions are weakened, leading decision-makers to rely more heavily on System 1. Consequently, framing-consistent intuitive responses are more likely to be directly expressed in choice behavior, resulting in stronger observed framing effects (Diederich & Trueblood, 2018; Diederich et al., 2020; Roberts et al., 2022).

However, systematic research in recent years indicates that, although time pressure does alter risk preferences, the direction of its effects is not uniform (Kühberger, 2023; Wyszynski & Diederich, 2023). A meta-analysis spanning four decades of research revealed substantial heterogeneity in the effects of time pressure on risky decision-making, with outcomes being jointly moderated by factors such as task characteristics and outcome framing (Belli et al., 2023). Some studies have found that time pressure increases risk-seeking tendencies (Phillips-Wren & Adya, 2020; Lin & Jia, 2023; Saqib & Chan, 2015), attributing this pattern to a greater reliance on fast heuristic judgments under urgency. Other studies, however, have reported limited effects of time pressure or even decreases in risk taking and attenuated framing effects (Haji et al., 2019; Olschewski & Rieskamp, 2021; Philips et al., 2025; Szaszi et al., 2022; Svenson & Benson, 1993). Recent evidence further suggests that canonical shifts in risk preference are more likely to emerge when sufficient decision time is available (Almeida & Rodrigues, 2024). Despite these inconsistencies, there is broad consensus that time pressure alters decision processes by constraining cognitive resources and processing depth (Gathmann et al., 2014; Jiang et al., 2024; Jocham et al., 2014; Zhou et al., 2024). Under high time pressure, increased cognitive load impairs systematic comparison and deliberative reasoning, leading individuals to rely more heavily on limited cues and to respond rapidly based on experience or intuition, thereby increasing susceptibility to biased or non-rational preferences (Bobadilla-Suarez & Love, 2018; Frank et al., 2009; Young et al., 2012; Verplanken, 1993). Importantly, recent studies employing electroencephalography (EEG) and functional magnetic resonance imaging (fMRI) further indicate that the brain does not “stop thinking” under time pressure but instead processes information at an accelerated pace (S. Li et al., 2025), suggesting that time pressure alters the tempo rather than the willingness to engage in cognitive processing.

Given the evidence that time pressure may alter risk preferences by weakening deep, deliberative processing, a critical question arises: under conditions of limited cognitive resources, what information do individuals actually rely on when making judgments? Cognitive Resource Theory provides an important framework for addressing this issue. According to this theory, attention is a limited cognitive resource (Kahneman, 1973), and time pressure, as an external stressor, further depletes the resources available for fine-grained processing, thereby promoting more economical information-processing strategies. When decision-makers face complex tasks or severe time constraints, they are often unable to systematically weigh all available information; instead, they prioritize more critical or salient cues while reducing the processing of secondary information (Svenson & Edland, 1987; Teoh & Hutcherson, 2022). Building on this account, Fuzzy-Trace Theory further elucidates the nature of information processing under resource constraints. This theory proposes that individuals form two parallel types of mental representations when making decisions under risk: precise, verbatim representations and fuzzy, gist-based representations (Reyna & Brainerd, 1991). When cognitive resources are sufficient, individuals can rely on verbatim representations to engage in analytical and quantitative evaluation. Under time pressure or high task demands, however, limited resources lead individuals to depend more on fast and effort-saving gist-based processing (Reyna et al., 2018). This mode of processing prioritizes the categorical meaning and emotional salience of outcomes over precise numerical calculations. Specifically, decision-makers extract the overall valence (positive or negative) of an option and respond accordingly. For example, a certain loss is represented as a salient, negatively valenced outcome, which motivates risk-seeking behavior to avoid the loss, whereas a certain gain is represented as a salient, positively valenced outcome, which motivates risk-averse behavior to secure the gain. Thus, gist-based processing is highly sensitive to the certainty of outcomes, and the resulting choices are determined by the emotional valence of that certainty rather than by the objective numerical magnitude or expected value of the option. Consequently, time pressure effectively shifts decision-making from “calculation” to “intuition”, and this shift constitutes a key cognitive mechanism through which framing effects are amplified (Pieters & Warlop, 1999).

Between the constraints on cognitive resources and the reliance on gist-based representations, strategic allocation of visual attention constitutes a critical behavioral bridge. Although traditional dual-system models can account for the tendency toward stronger framing effects under time pressure, emerging evidence from the visual attention literature suggests that this explanation may be incomplete. An increasing number of studies indicate that time pressure redirects early attentional orientation and alters how attentional resources are distributed among task-relevant cues (Hu et al., 2023; Roberts et al., 2022). Visual attention has been shown to be a stable predictor of risk preferences and risky choices (Bhatnagar & Orquin, 2022), and eye-tracking methods, which capture attentional dynamics in real time, have, therefore, become a critical tool for examining the interaction between time pressure and risky decision-making (Horstmann et al., 2009; Wei et al., 2025). Eye-tracking evidence suggests that individuals under time pressure do not passively become “irrational” but instead actively adopt more adaptive information-search strategies. Specifically, they tend to direct early attention more rapidly toward key information related to gains or losses, particularly toward certain options that are most easily distilled into gist-based representations, while reducing the processing of compensatory cues and terminating information search earlier (Roberts et al., 2022; Wedel et al., 2022; Zhou et al., 2024). This strategic allocation of attention reflects a redistribution of cognitive resources and reliably predicts subsequent choice behavior. Hu et al. (2023) further demonstrated that gaze patterns in risky decision-making unfold in stages: early fixations are concentrated on highly salient or emotionally charged cues, whereas later stages involve integration and trade-off processes. Under time constraints, this early attentional bias is markedly amplified, such that preferences are often formed at the very first glance. Compared with self-report measures or response times, eye-tracking metrics provide a more sensitive window into underlying information-processing dynamics (Mueller et al., 2022). Converging evidence shows that, under time pressure, individuals exhibit a significantly increased number of fixations and dwell time on certain options, accompanied by reduced exploration of risky alternatives (Guo et al., 2017; Wei et al., 2025), indicating a stronger reliance on certainty-related cues and faster preference formation when cognitive resources are limited. Accordingly, eye-tracking evidence not only offers direct support for the proposed time pressure–attention allocation–framing effect pathway but also provides micro-level validation for the processing shift posited by Fuzzy-Trace Theory. By integrating behavioral choices with eye-tracking measures, the present study aims to further elucidate the cognitive foundations of risky decision-making under time pressure and the dynamic role of visual attention in this process.

Building on the attentional mechanisms outlined above, the present study further focuses on the role of external visual presentation factors in strategic attention allocation. A substantial body of empirical research indicates that the spatial layout and presentation order of stimuli within the visual field can alter individuals’ information-search paths, thereby influencing attentional resource allocation and risk preferences (Gluth et al., 2020; Orquin et al., 2021). Early studies demonstrated that spatial position significantly affects information processing. For example, Pieters and Warlop (1999) showed that, under time pressure, individuals are more susceptible to left-to-right reading habits, preferentially processing information that appears earlier along the visual scanning path and maintaining this initial bias in subsequent choices. Similarly, Zuschke (2023) reported that options occupying visually advantageous positions receive earlier and more sustained attention, indicating that spatial position amplifies asymmetries in attention allocation. Subsequent research has further shown that presentation order can also shape preference formation. Kwak and Huettel (2018) demonstrated that information presented earlier is more likely to attract early fixations, a process that amplifies framing effects. Specifically, early fixations grant priority to initially attended framing information (e.g., a “sure gain” or a “sure loss”), increasing its likelihood of being encoded as a salient gist representation that sets the initial affective context for the decision. Under time pressure, limited cognitive resources reduce the extent to which alternative options can be fully evaluated, thereby increasing the probability that decisions rely disproportionately on this early-encoded gist. Consequently, framing-consistent information that is fixated early exerts a stronger influence on choice behavior, resulting in stronger observed framing effects. Millroth et al. (2019) demonstrated that sequential, as opposed to simultaneous, presentation of probabilities and outcomes alters risk evaluation. Broader decision-making research has also shown that the order in which information is inspected systematically influences visual attention allocation and the evaluation of alternatives (Heard et al., 2018). Roberts et al. (2022) and Hu et al. (2023) further demonstrated that early attentional biases and premature termination of information search are key mechanisms underlying the amplification of framing effects under time pressure. Taken together, this evidence suggests that the strengthening of framing effects under time pressure may not be driven solely by constrained cognitive resources but is jointly moderated by visual presentation factors such as spatial position and presentation order. When certain options occupy visually advantageous positions or are presented earlier, individuals are more likely to form initial preferences at an early stage and, under time constraints, rely on these preferences when making choices.

Grounded in Cognitive Resource Theory and Fuzzy-Trace Theory, time pressure constrains the opportunity for extended verbatim and quantitative processing, thereby promoting a strategic shift toward gist-based representations that efficiently capture the essential meaning of options and increasing sensitivity to highly salient cues at early stages of processing. Consistent with prior eye-tracking findings, individuals under time constraints tend to adopt strategic patterns of attention allocation. At the same time, visual presentation factors, such as the spatial position and presentation order of options, can systematically guide attentional trajectories and influence risky choice. Accordingly, the present research comprised two sequential experiments. Study 1 primarily examined the effects of time pressure on framing effects and attention allocation, with spatial position included as a potential moderating factor. Study 2 further manipulated the presentation order of options to examine how spatial position and presentation order may interact with time pressure in shaping attention allocation and risky decision-making. In addition to our primary hypotheses regarding the moderating role of time pressure on framing effects, spatial position (Study 1) and presentation order (Study 2) were incorporated to examine potential higher-order interactions. Given limited prior evidence specifying directional predictions for these interactions, no a priori directional hypotheses were proposed.

## 2. Experiment 1

This study employed eye-tracking technology to investigate how time pressure and option position influence the framing effect in risk decision-making. Drawing upon the Cognitive Resource Theory of Attention and the Fuzzy-Trace Theory, the experiment aimed to examine how individuals allocate attention to different options under constrained conditions. By analyzing behavioral choices, reaction times, and key eye-tracking indicators (e.g., fixation duration, fixation counts, first fixations), this study sought to elucidate the mechanisms of attentional deployment and the cognitive processes underlying the framing effect. Specifically, based on prior research on time pressure and framing, we expected that time pressure would amplify the framing effect in risky decision-making by increasing risk seeking in loss-framed contexts and risk aversion in gain-framed contexts. At the attentional level, time pressure was expected to bias visual attention toward certainty-related information, as reflected in longer fixation durations and a greater number of fixations on the certain option. Regarding spatial position, although its effects may be attenuated by the fully randomized trial design, it was systematically examined as a factor interacting with time pressure, with particular attention to its role in shaping early visual attention and moderating the framing effect.

### 2.1. Method

#### 2.1.1. Participants

Thirty college students (13 males, 17 females) participated in the experiment. All participants had normal or corrected-to-normal vision, were right-handed, and reported no history of reading or neurological impairments. An a priori power analysis was conducted using G*Power 3.1.9 (Faul et al., 2007) for a repeated-measures ANOVA (within factors). Consistent with prior studies employing multi-factor within-subject designs (He & Yuan, 2025), the effect size was set to *f* = 0.25 (medium), α = 0.05, and desired power (1 − β) = 0.80. The study employed a three-factor, two-level within-subjects design and primarily examined whether the effect of framing on risky decision-making varied as a function of time pressure and spatial position. The power analysis indicated a minimum required sample size of 15 participants. All participants provided written informed consent prior to participation. After data screening, four participants were excluded due to unsuccessful eye-tracker calibration or excessive trial omissions, resulting in a final sample of 26 participants included in the analyses.

#### 2.1.2. Experimental Design

A 2 (Time Pressure: 1 s, No Time Pressure) × 2 (Frame: Loss, Gain) × 2 (Position: Certain Option Left, Certain Option Right) within-subjects design was employed. The dependent variables included both behavioral and eye-tracking measures. Behavioral measures comprised the proportion of risky choices and decision reaction times. Eye-tracking measures were further categorized into attention initiation indices and attention maintenance indices. Attention initiation was operationalized as the type of option receiving the first fixation, indexing early attentional orientation. Attention maintenance was indexed by fixation count and total fixation duration on each option, reflecting the depth of information processing and the allocation of cognitive resources.

#### 2.1.3. Experimental Materials

**Figure 1:**
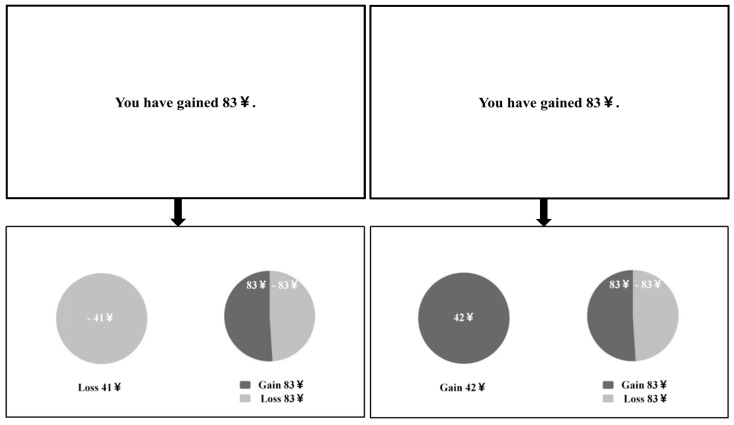
Example diagram of experimental materials.

The experimental materials were adapted from the risky choice task developed by Guo et al. (2017). The experiment included 72 unique “initial amount–probability” combinations. Initial amounts were randomly drawn from a uniform distribution, U(20, 90), and probabilities of winning the gamble were generated from a truncated normal distribution. For each combination, participants chose between a certain option and a gamble (risky) option. The expected value of the certain option was matched to that of the gamble option for each trial. Based on each combination, both gain-framed and loss-framed versions of the decision problem were constructed, resulting in two equivalent framing versions of the same choice problem. Critically, the gamble option was identical across gain and loss frames; the framing manipulation applied only to the certain option. For example, when the initial amount was 60 yuan and the probability of winning the gamble was 0.25, the gamble involved a 0.25 probability of keeping the full amount and a 0.75 probability of losing it. The certain option was framed as “Keep 15 yuan” in the gain frame and as “Lose 45 yuan” in the loss frame, yielding equivalent expected values across frames. Gain and loss information in the stimuli was indicated using different colors and was counterbalanced across conditions. Stimulus text was presented in bold font (24 pt), with white text on a black background, on a 19-inch monitor with a resolution of 1024 × 768. All experimental procedures were programmed and presented using the built-in software of the SR Research EyeLink 1000 Plus eye-tracking system. Eye-movement data were collected using a desktop-mounted eye tracker at a sampling rate of 2000 Hz. Participants were seated approximately 50 cm from the display. Although binocular eye movements were recorded throughout the experiment, only right-eye gaze data were included in the analyses. The examples of materials are shown in Figure 1.

#### 2.1.4. Experimental Procedure

The study was approved by the relevant Research Ethics Committee and conducted in accordance with established ethical standards. Upon arrival at the laboratory, participants provided informed consent and received instructions regarding the risk choice task and the eye-tracking procedure. The experimenter ensured that participants fully understood the task requirements and the eye-tracking process, including head stabilization and fixation on calibration points.

The experiment included two time conditions: no time pressure and time pressure (1 s). Each participant completed both conditions, with the order counterbalanced across participants. Prior to each condition, participants completed 32 practice trials to become familiar with the task rules, timing, and response procedures. Each time condition consisted of 144 formal trials, yielding a total of 288 formal trials across the experiment. The 144 trials in each condition were based on the same 72 unique initial amount–probability combinations, with each combination presented once in the gain frame and once in the loss frame, and these gain- and loss-framed trials were randomly intermixed within each condition. All trials were further divided into four blocks of 72 trials, with short rest breaks between blocks.

At the beginning of each trial, the initial amount (e.g., “You have 83 yuan”) was displayed for 2 s. This was followed by the simultaneous presentation of the certain option and the gamble option. Across all trials and conditions, the left–right positions of the sure and gamble options were counterbalanced, such that the certain option appeared equally often on the left and right for each participant, while the order of trials within each block was fully randomized. In the no-time-pressure condition, participants were instructed to “Please consider carefully and choose the option you believe will maximise your payoff”, and no time limit was imposed on responding. In the time-pressure condition, participants were instructed to “Please respond as quickly as possible within one second”, and each decision had to be made within a 1000 ms deadline. Although the explicit instructions differed in emphasis (accuracy vs. speed), both conditions required participants to make outcome-based choices and accumulate points across trials. Specifically, the manipulation primarily targeted processing time while preserving the same payoff structure across conditions. Following established paradigms in research on risky decision-making under time constraint (Guo et al., 2017; Roberts et al., 2022), we implemented a differential feedback structure to balance speed and accuracy while maintaining task motivation. In the no-time-pressure condition, feedback indicating the number of points earned on each trial was presented after every response. In the time-pressure condition, feedback was presented only when participants failed to respond before the deadline, indicating that no points were earned due to a timeout; timely responses did not receive trial-by-trial feedback. This structure implicitly incentivized participants under time pressure to respond both quickly, in order to avoid timeouts, and accurately, in order to obtain favorable outcomes associated with their chosen options. Throughout the task, cumulative points served as ongoing performance feedback but were not directly tied to monetary bonuses. Although no monetary bonus was tied to performance, participants were informed that cumulative points reflected their task performance, consistent with widely used hypothetical risky choice paradigms.

**Figure 2:**
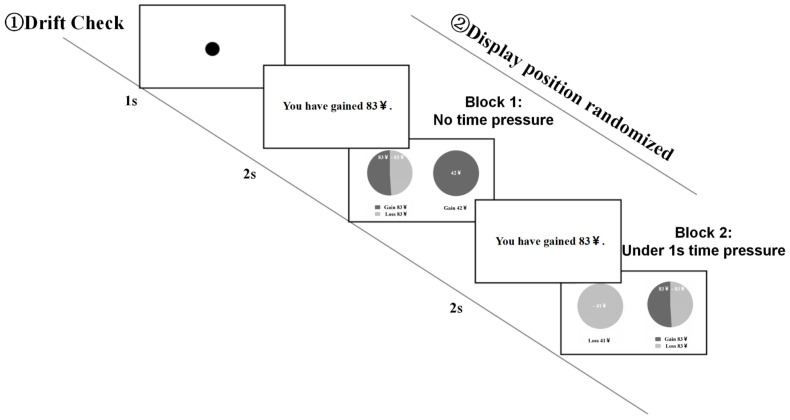
Measurement flow chart of Experiment 1.

Participants indicated their choices by pressing the Z key for the left option and the M key for the right option. Each trial terminated immediately after a response was made. The full experiment, including calibration, practice, and rest periods, lasted approximately 30 min. A schematic of the experimental procedure is shown in Figure 2.

#### 2.1.5. Data Processing

Eye-tracking data were collected using the EyeLink 1000 Plus system and analyzed with SPSS 26.0, PROCESS v3.3, and Data Viewer 4.3.1. Prior to analysis, data were screened according to the following exclusion criteria: (1) uncorrected visual impairments; (2) under the no-time-pressure condition, selecting the lower-value option in more than 25% of mismatched expected-value trials; (3) choosing the same option (e.g., risky or certain) in over 90% of all trials; (4) failing to respond in more than 25% of trials under time pressure; and (5) poor eye-tracker calibration quality. Based on these criteria, four participants were excluded from further analysis. To examine the effects of time pressure, frame, and spatial position on risky decision-making, all analyses were conducted using repeated-measures ANOVA.

### 2.2. Results

#### 2.2.1. Behavioral Results: Risky Choice Proportion

To examine the effect of time pressure on the framing effect in risky decision-making, a 2 (Time Pressure: 1 s vs. no time pressure) × 2 (Frame: gain vs. loss) repeated-measures ANOVA was conducted on the proportion of risky choices (see Table 1). The main effect of frame was significant, *F*(1, 25) = 282.58, *p* < 0.001, $\eta_{p}^{2}$ = 0.92, with a higher proportion of risky choices in the loss frame than in the gain frame. The main effect of time pressure was also significant, *F*(1, 25) = 7.55, *p* < 0.05, $\eta_{p}^{2}$ = 0.23. The interaction between time pressure and frame was significant, *F*(1, 25) = 309.50, *p* < 0.001, $\eta_{p}^{2}$ = 0.93. Simple-effects analyses showed that, in the gain frame, the proportion of risky choices was lower under time pressure (*M* = 0.11, *SD* = 0.09) than under no time pressure (*M* = 0.36, *SD* = 0.11), whereas in the loss frame, the proportion of risky choices was higher under time pressure (*M* = 0.85, *SD* = 0.15) than under no time pressure (*M* = 0.49, *SD* = 0.10). These results indicate that time pressure not only amplified the framing effect but also reversed the direction of risk preference depending on the frame.

To examine the potential moderating role of spatial position on the interaction between time pressure and frame, a 2 (Time Pressure) × 2 (Frame) × 2 (Position: Certain Option Left vs. Certain Option Right) repeated-measures ANOVA was conducted (see Table 1). The main effect of frame was significant, *F*(1, 25) = 117.92, *p* < 0.001, $\eta_{p}^{2}$ = 0.83, and the main effect of position was significant, *F*(1, 25) = 6.32, *p* < 0.05, $\eta_{p}^{2}$ = 0.20. The interaction between time pressure and frame was also significant, *F*(1, 25) = 64.72, *p* < 0.001, $\eta_{p}^{2}$ = 0.72. However, none of the two-way or three-way interactions involving position reached significance (*ps* > 0.05). This pattern indicates that spatial position did not significantly moderate the effect of time pressure on framing under the current experimental conditions.

To further characterize the position effect under no time pressure, a further 2 (Position: Certain Option Left vs. Certain Option Right) × 2 (Frame: gain vs. loss) repeated-measures ANOVA was conducted on trials without time pressure (see Table 1). The main effect of position was significant, *F*(1, 25) = 9.74, *p* < 0.01, $\eta_{p}^{2}$ = 0.28, and the main effect of frame was also significant, *F*(1, 25) = 35.63, *p* < 0.001, $\eta_{p}^{2}$ = 0.59. The interaction between position and frame was significant, *F*(1, 25) = 6.07, *p* < 0.05, $\eta_{p}^{2}$ = 0.20. Simple-effects analyses revealed that, in the loss frame, participants were more likely to choose the risky option when the certain option was presented on the left (*M* = 0.69, *SD* = 0.05) than on the right (*M* = 0.57, *SD* = 0.06). In the gain frame, the position effect was not significant (Left: *M* = 0.37, *SD* = 0.05; Right: *M* = 0.33, *SD* = 0.05). This interaction reflects a frame-dependent position effect that emerged only in the absence of time pressure.

#### 2.2.2. Behavioral Results: Reaction Time Analysis

A 2 (Time Pressure: 1 s vs. no time pressure) × 2 (Frame: gain vs. loss) repeated-measures ANOVA was conducted on reaction times. The main effect of time pressure was significant, *F*(1, 25) = 51.98, *p* < 0.001, $\eta_{p}^{2}$ = 0.68, indicating that time constraints significantly accelerated decision-making. The main effect of frame was also significant, *F*(1, 25) = 8.44, *p* < 0.01, $\eta_{p}^{2}$ = 0.25, with participants taking longer to decide in the loss frame than in the gain frame. The interaction between time pressure and frame was significant, *F*(1, 25) = 8.94, *p* < 0.01, $\eta_{p}^{2}$ = 0.26. Simple-effects analyses revealed that, under time pressure, reaction times did not differ significantly between the gain frame (*M* = 654.49, *SD* = 23.05) and the loss frame (*M* = 680.74, *SD* = 24.26). In contrast, under no time pressure, reaction times were significantly shorter for the gain frame (*M* = 1997.73, *SD* = 190.51) than for the loss frame (*M* = 2548.90, *SD* = 287.12). These results indicate that time pressure reduces decision latency and that participants require more time to process decisions in the loss frame, particularly when no time constraints are imposed.

#### 2.2.3. Eye-Tracking Results: Early Attentional Orientation

**Figure 3:**
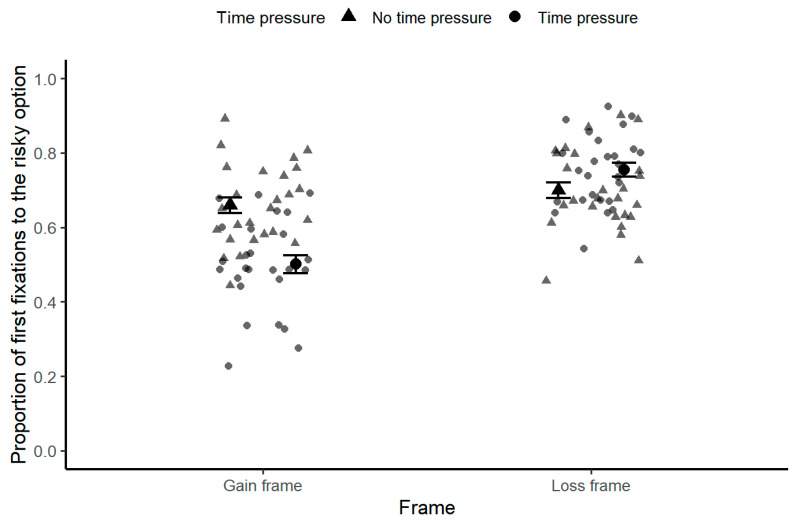
First fixations on the risky option across time pressure and frame conditions. Mean proportion of first fixations directed toward the risky option under no-time-pressure (circles, white fill) and time-pressure (triangles, gray fill) conditions, separately for gain and loss frames. Error bars represent ±1 standard error of the mean (N = 26).

A 2 (Time Pressure: 1 s vs. no time pressure) × 2 (Frame: gain vs. loss) repeated-measures ANOVA was conducted on the proportion of first fixations directed toward the risky option (see Figure 3). The main effect of time pressure was significant, *F*(1, 25) = 4.53, *p* < 0.05, $\eta_{p}^{2}$ = 0.15, with a lower proportion of initial fixations on the risky option under time pressure. The main effect of frame was also significant, *F*(1, 25) = 72.47, *p* < 0.001, $\eta_{p}^{2}$ = 0.74, with a higher proportion of first fixations on the risky option in the loss frame than in the gain frame. The interaction between time pressure and frame was significant, *F*(1, 25) = 25.55, *p* < 0.001, $\eta_{p}^{2}$ = 0.51. Simple-effects analyses showed that, in the gain frame, participants were less likely to direct their first fixation toward the risky option under time pressure (*M* = 0.50, *SD* = 0.02) than under no time pressure (*M* = 0.66, *SD* = 0.02). In the loss frame, no significant difference was found between time-pressure conditions. This interaction indicates that time pressure altered early attentional orientation toward risky options in a frame-dependent manner. The implications of these early attentional patterns for framing-consistent choice behavior are discussed in the discussion.

#### 2.2.4. Eye-Tracking Results: Sustained Attention Allocation

**Figure 4:**
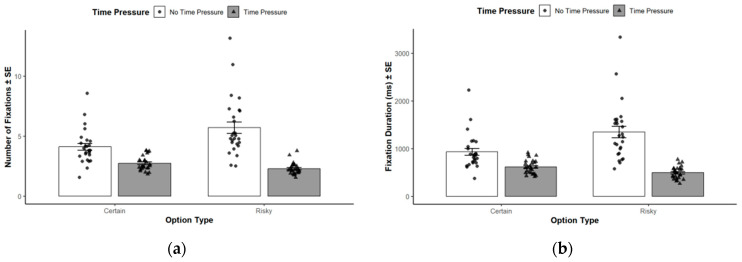
Eye movement measures across option types and time-pressure conditions. (**a**) Mean number of fixations and (**b**) mean fixation duration (ms) to certain and risky options under no-time-pressure (white bars) and time-pressure (gray bars) conditions. Error bars represent ±1 standard error of the mean (*N* = 26). Individual data points are shown with circles (no time pressure) and triangles (time pressure).

Based on Cognitive Resource Theory, this analysis focuses on the effect of time pressure on the allocation of resources during deep processing; data were aggregated across framing conditions, and framing effects were assessed using the first fixation proportion measure. To further examine attentional allocation over time, a 2 (Time Pressure: 1 s vs. no time pressure) × 2 (Option Type: certain vs. risky) repeated-measures ANOVA was conducted on fixation count and fixation duration (see Figure 4).

For fixation count, the main effect of time pressure was significant, *F*(1, 25) = 47.32, *p* < 0.001, $\eta_{p}^{2}$ = 0.65, indicating fewer fixations under time pressure. The main effect of option type was also significant, *F*(1, 25) = 20.10, *p* < 0.001, $\eta_{p}^{2}$ = 0.45, with more fixations on risky options overall. The interaction between time pressure and option type was significant, *F*(1, 25) = 42.87, *p* < 0.001, $\eta_{p}^{2}$ = 0.63. Simple-effects analyses indicated that, under time pressure, participants made more fixations on certain options (*M* = 2.73, *SD* = 0.12) than on risky options (*M* = 2.28, *SD* = 0.10). In contrast, under no time pressure, fixation counts were higher for risky options (*M* = 5.70, *SD* = 0.48) than for certain options (*M* = 4.11, *SD* = 0.28).

A parallel pattern was observed for fixation duration. The main effect of time pressure was significant, *F*(1, 25) = 43.94, *p* < 0.001, $\eta_{p}^{2}$ = 0.64, with shorter fixation duration under time pressure. The main effect of option type was significant, *F*(1, 25) = 23.29, *p* < 0.001, $\eta_{p}^{2}$ = 0.48, reflecting longer fixation duration on risky options. The interaction between time pressure and option type was also significant, *F*(1, 25) = 50.62, *p* < 0.001, $\eta_{p}^{2}$ = 0.67. Specifically, under time pressure, fixation duration was longer for certain options (*M* = 615.93, *SD* = 29.35) than for risky options (*M* = 502.39, *SD* = 24.12). Under no time pressure, this pattern reversed, with longer fixation duration for risky options (*M* = 1349.58, *SD* = 119.22) than for certain options (*M* = 933.23, *SD* = 72.86).

These findings characterize a reversal in sustained attention allocation across option types as a function of time pressure. The implications of this pattern for decision-making under time constraints are discussed below.

### 2.3. Discussion

The present study examined how time pressure and spatial position were associated with framing effects in risky decision-making. Behavioral results showed that time pressure significantly amplified framing effects. Under time constraints, individuals were more likely to choose the certain option in the gain frame to secure a sure gain and the risky option in the loss frame to avoid a sure loss compared to the no-pressure condition. This pattern suggests that time pressure may increase reliance on frame-consistent aspects of the options, which may reflect that they may focus on securing sure gains in the gain frame and avoiding sure losses in the loss frame, potentially through meaning-based (gist) processing when forming decisions. Eye-tracking findings revealed stage-specific attentional effects under time pressure. During early attention, indexed by first fixations, participants showed greater initial focus on the risky option in the loss frame than in the gain frame, and time pressure reduced first fixations on the risky option in the gain frame only. During later processing, reflected in fixation counts and total fixation duration aggregated across frames, time pressure was associated with reduced overall information search and relatively greater attention to the certain option. These attentional patterns are consistent with the possibility that time constraints could selectively modulate early attention in a frame-specific manner and be associated with shifts in attentional allocation during later processing toward options providing more immediately interpretable, frame-consistent outcomes, potentially supporting reliance on the frame-consistent salient gist of options through meaning-based (gist) processing (Roberts et al., 2022; Hu et al., 2023). Regarding spatial presentation, the influence of option position on risky decision-making was relatively limited under the present randomized design. Option locations were counterbalanced on a trial-by-trial basis to control for systematic spatial biases. While this procedure enhances internal validity, it may reduce the stability of spatial biases across trials compared to a blocked spatial design that allows positional biases to manifest more consistently, thereby possibly attenuating the statistical power to detect a robust position effect. Under no time pressure, however, the interaction between position and frame was significant. Specifically, in the loss frame, when the certain option was presented on the left, individuals were more likely to choose the risky option. This pattern may reflect habitual reading or scanning tendencies, whereby left-sided information could gain an advantage during early attentional deployment and subsequently may shape downstream decision tendencies (Pieters & Warlop, 1999; Orquin et al., 2021). In contrast, under time pressure, position effects were not significant, suggesting that temporal constraints may influence attentional configuration, compress information search paths, and attenuate the influence of spatial layout on decision-making (Lin & Jia, 2023; Zhou et al., 2024). Overall, Study 1 indicates that, within the present design, time pressure exerted a statistically stronger influence on framing-related choice patterns than spatial position. Rather than uniformly shifting risk preference, time pressure was associated with changes in how participants allocated attention across options and frames. These findings provide initial evidence that temporal constraints may shape the expression of framing effects, while the role of spatial position appears more limited and context-dependent.

## 3. Experiment 2

Study 1 demonstrated that time pressure significantly amplifies the framing effect and revealed, through eye-tracking measures, that individuals under time constraints tend to focus attention on certainty cues, reflecting a reallocation of attentional resources. However, Study 1 primarily focused on the effects of time pressure and spatial position, whereas real-world decision-making is often influenced by the temporal order in which information is presented. The sequence of incoming information can change its attentional priority and, consequently, shape final choices. Prior research suggests that time pressure compresses information processing and magnifies sequence-based effects, leading to differential attentional weighting depending on the temporal order of information presentation (Perkovic et al., 2023; Y. R. Li & Liu, 2024). Building on these findings, Study 2 introduced the presentation order variable to further examine how time pressure, spatial position, and presentation sequence jointly influence the framing effect. This design aimed to capture the integrated influence of temporal and spatial dimensions on attentional mechanisms in risk decision-making. It was hypothesized that, under time pressure, individuals would rely more heavily on earlier-presented information, leading to stronger sequential biases. Meanwhile, spatial position and presentation order were expected to interactively shape risk preferences by guiding attention allocation. Considering evidence that spatial effects may stem from sequential information processing (Kwak & Huettel, 2018), this study investigates how dynamic shifts in visual attention and temporal constraints jointly determine framing effects in contexts that more closely resemble real-world decision scenarios. Specifically, it examines how time pressure, spatial position, and presentation order interact in shaping the framing effect in risky choice.

### 3.1. Method

#### 3.1.1. Participants

Thirty college students (13 males, 17 females) participated in this study. All participants had normal or corrected-to-normal vision, were right-handed, and reported no reading or neurological impairments. An a priori power analysis was conducted using G*Power 3.1.9 (Faul et al., 2007) for a repeated-measures ANOVA (within factors). Following prior research employing multi-factor within-subject designs (He & Yuan, 2025), the effect size was set to *f* = 0.25, α = 0.05, and desired power (1 − β) = 0.80. The study adopted a four-factor, two-level within-subjects design, and the experiment primarily examined whether presentation order moderated framing effects under different levels of time pressure. Spatial position was included as an exploratory factor. The power analysis indicated a minimum required sample size of 11 participants. All participants provided written informed consent prior to the experiment. Six participants were excluded due to failed eye-tracker calibration or excessive omission rates, resulting in a final sample of 24 valid datasets for analysis.

#### 3.1.2. Experimental Design

A 2 (Time Pressure: 1 s, No Time Pressure) × 2 (Frame: Loss, Gain) × 2 (Presentation Order: Certain Option First, Certain Option Second) × 2 (Position: Certain Option Left, Certain Option Right) within-subjects design was employed. The primary dependent variable in Study 2 was a behavioral measure: the proportion of risky choices, which served as the core index of the framing effect.

#### 3.1.3. Experimental Materials

The materials and visual stimuli in Experiment 2 were identical to those used in Experiment 1, including the gain and loss framing tasks. Across trials, the certainty and risky options were randomly presented on the left or right side of the screen, with spatial position fully counterbalanced.

#### 3.1.4. Experimental Procedure

**Figure 5:**
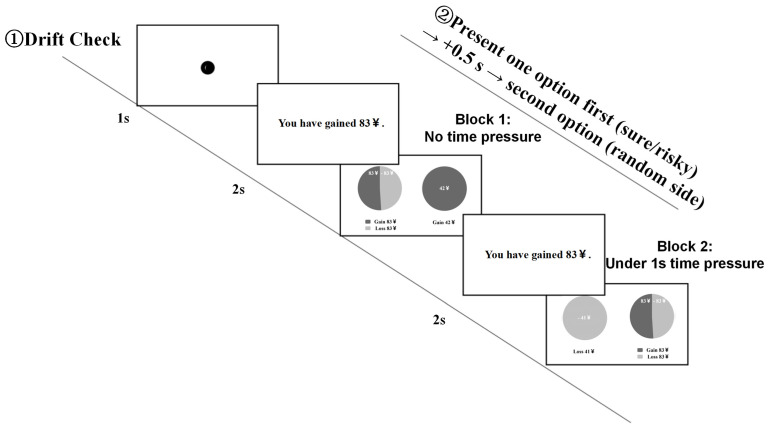
Measurement flow chart of Experiment 2.

The main task procedure of Experiment 2 closely followed that of Experiment 1, with one critical modification introduced to examine the effect of presentation order. In half of the trials, the certainty option was presented first, followed by the risky option after a delay of 0.5 s (Giesbrecht & Di Lollo, 1998; Isaak et al., 1999). In the remaining trials, the presentation order was reversed, with the risky option appearing first and the certainty option presented after the same delay. Once both options were displayed, they remained visible on the screen until the participant made a response. All other parameters, including display resolution, color scheme, font, and presentation duration, were kept identical to those in Experiment 1. A schematic of the experimental procedure is presented in Figure 5.

#### 3.1.5. Data Processing

Data collection and preprocessing procedures were identical to those used in Study 1. To test the study hypotheses, all analyses were conducted using repeated-measures ANOVA.

### 3.2. Results

Given the complexity of the higher-order interactions and the exploratory nature of the presentation-order and spatial-position manipulations, interpretation of these effects is deferred to the discussion section.

#### 3.2.1. Four-Factor Repeated-Measures ANOVA

To systematically examine the joint effects of time pressure, frame, presentation order, and spatial position on risky decision-making, a four-factor repeated-measures ANOVA was conducted on the proportion of risky choices, with 2 (Time Pressure: 1 s vs. No Time Pressure) × 2 (Frame: Gain vs. Loss) × 2 (Presentation Order: Certain Option First vs. Certain Option Second) × 2 (Position: Certain Option Left vs. Right) as within-subject factors.

The results revealed a significant main effect of frame, *F*(1, 23) = 32.72, *p* < 0.001, $\eta_{p}^{2}$ = 0.59, such that the proportion of risky choices was significantly higher in the loss frame than in the gain frame. The main effect of presentation order was also significant, *F*(1, 23) = 8.02, *p* < 0.01, $\eta_{p}^{2}$ = 0.26, indicating a higher likelihood of choosing the risky option when the certain option was presented second rather than first. The main effects of time pressure and position were not significant (*ps* > 0.30).

Regarding two-way interactions, the time pressure × presentation order interaction was significant, *F*(1, 23) = 27.85, *p* < 0.001, $\eta_{p}^{2}$ = 0.55. In addition, the presentation order × position interaction reached significance, *F*(1, 23) = 4.95, *p* < 0.05, $\eta_{p}^{2}$ = 0.18. All remaining two-way interactions were not significant (*ps* > 0.10).

Critically, the time pressure × frame × presentation order three-way interaction was significant, *F*(1, 23) = 13.58, *p* < 0.01, $\eta_{p}^{2}$ = 0.37, indicating that the joint influence of frame and presentation order on risky choice was significantly moderated by time pressure. All other three-way and the four-way interactions were not significant (*ps* > 0.30). Overall, the results indicate that framing effects were robust, while the influence of presentation order on risky choice was contingent on time pressure and, to a lesser extent, on spatial position. The complete ANOVA results are reported in Table 2.

#### 3.2.2. Decomposition of Significant Interactions

##### Time Pressure × Presentation Order Interaction

**Figure 6:**
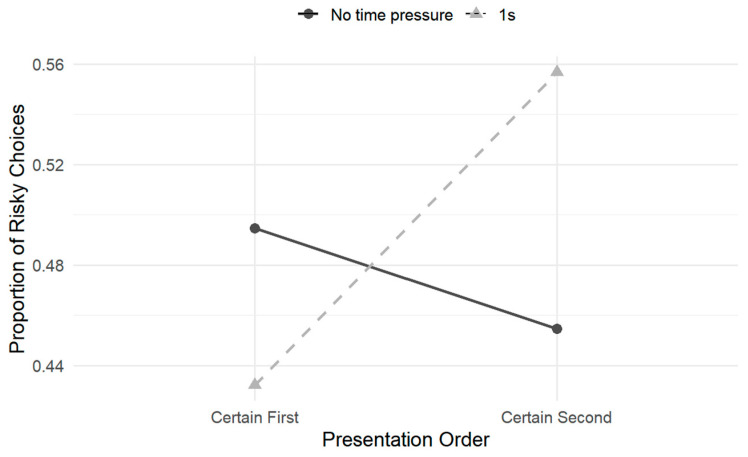
Interaction between time pressure and presentation order on risky choice proportion (adjusted means).

To further interpret the significant time pressure × presentation order interaction, simple-effects analyses were conducted to examine the effect of presentation order separately under each time-pressure condition (see Figure 6). The results showed that, under time pressure, the proportion of risky choices was significantly higher when the certain option was presented second (*M* = 0.54, *SD* = 0.03) than when it was presented first (*M* = 0.41, *SD* = 0.03), *t* = 4.58, *p* < 0.001. In contrast, under no time pressure, the direction of the presentation order effect was reversed: participants were more likely to choose the risky option when the certain option was presented first (*M* = 0.50, *SD* = 0.03) rather than second (*M* = 0.46, *SD* = 0.04), *t* = −2.87, *p* < 0.01. This interaction reflects a reversal in the effect of presentation order as a function of time pressure, with opposite choice patterns emerging under time-constrained versus unconstrained conditions.

##### Time Pressure × Frame × Presentation Order Three-Way Interaction

**Figure 7:**
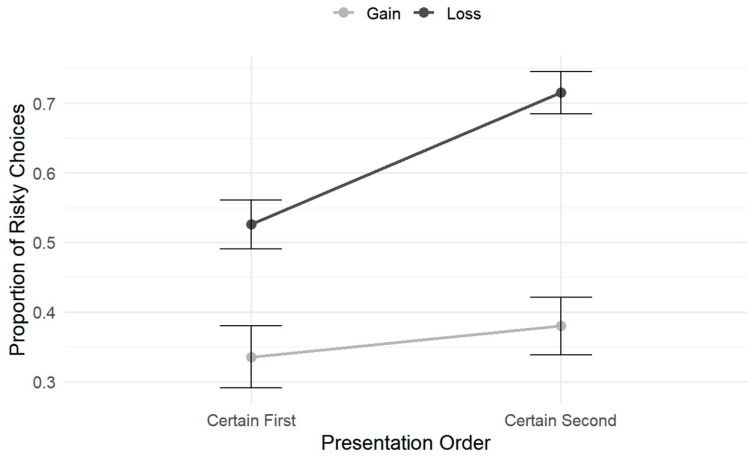
Interaction between framing and presentation order under time pressure. Participants showed greater risk preference when the certain option appeared second, particularly in the loss frame.

To decompose the significant three-way interaction, separate 2 (Frame) × 2 (Presentation Order) repeated-measures ANOVAs were conducted under the time-pressure and no-time-pressure conditions, respectively (see Figure 7).

Under time pressure, the main effect of frame was significant, *F*(1, 23) = 35.31, *p* < 0.001, $\eta_{p}^{2}$ = 0.61, and the main effect of presentation order was also significant, *F*(1, 23) = 21.01, *p* < 0.001, $\eta_{p}^{2}$ = 0.48. Importantly, the frame × presentation order interaction was significant, *F*(1, 23) = 11.32, *p* < 0.01, $\eta_{p}^{2}$ = 0.33. Simple-effects analyses indicated that, in the loss frame, the proportion of risky choices was significantly higher when the certain option was presented second (*M* = 0.71, *SD* = 0.03) than when it was presented first (*M* = 0.53, *SD* = 0.04), *t* = 5.68, *p* < 0.001. In contrast, in the gain frame, the effect of presentation order on risky choice did not reach significance, *t* = 1.82, *p* = 0.08.

Under no time pressure, the frame × presentation order interaction was not significant, *F*(1, 23) = 3.16, *p* = 0.09, $\eta_{p}^{2}$ = 0.12. However, both the main effect of frame, *F*(1, 23) = 20.79, *p* < 0.001, $\eta_{p}^{2}$ = 0.48, and the main effect of presentation order, *F*(1, 23) = 8.23, *p* < 0.01, $\eta_{p}^{2}$ = 0.26, remained significant.

Together, these results indicate that the combined influence of framing and presentation order on risky choice emerged primarily under time pressure, whereas under no time pressure, their effects did not interact and were expressed primarily as main effects.

##### Presentation Order × Position Interaction

To further examine the significant presentation order × position interaction, simple-effects analyses were conducted. The results showed that, when the certain option was presented on the right side, the presentation order effect was significant, with a higher proportion of risky choices when the certain option was presented second than when it was presented first, *t* = 4.80, *p* < 0.001. In contrast, when the certain option was presented on the left side, the presentation order effect was not significant, *t* = 0.88, *p* = 0.39. As this interaction did not involve time pressure or frame and was not central to the primary theoretical focus of the study, it was not examined further.

### 3.3. Discussion

Building on Experiment 1, Experiment 2 further manipulated the order of option presentation to examine whether framing effects under time pressure varied as a function of information order. The results revealed a significant interaction between time pressure and presentation order. Specifically, under time pressure, participants exhibited a stronger tendency toward risky choice when the certain option was presented second (i.e., when the risky option was presented first), whereas this pattern was reversed in the absence of time pressure. More importantly, a significant three-way interaction among time pressure, framing, and presentation order was observed. Follow-up simple-effects analyses showed that the influence of presentation order on risk preference varied across frames under time pressure. In particular, presenting the certain option second significantly increased risk preference in the loss frame, whereas this effect was not observed in the gain frame. No significant interaction between framing and presentation order was observed under no time pressure. Taken together, these findings suggest that the influence of time pressure on risky choice cannot be reduced to a uniform shift in overall risk preference but instead depends on how information is temporally presented. From a cognitive processing perspective, the pattern is consistent with the possibility that time pressure increases reliance on temporally ordered information rather than merely enhancing the salience of a particular option type. Under time constraints, asymmetries associated with presentation order may become more behaviorally consequential, potentially resembling an anchoring-like influence of early-presented information. In the present task, presenting the certain option second necessarily implies earlier exposure to the risky option. Thus, the increased risk preference observed under time pressure when the certain option was presented second may not reflect preferential processing of later-presented certain information. Rather, it may reflect a stronger initial evaluation formed from early exposure to the risky option under constrained cognitive resources, which could become more difficult to revise as the decision unfolds. When cognitive resources are limited by time pressure, individuals’ capacity to systematically integrate and reweigh subsequently presented information may be reduced, potentially leading early-formed judgments to become stabilized in the decision process. The effect was particularly evident in the loss frame, where risky options may be intuitively construed as a means of avoiding certain losses. Early exposure to such options may more readily reinforce this gist representation, thereby potentially amplifying risk-seeking tendencies under time pressure (Reyna & Brainerd, 1995; Reyna et al., 2018). Accordingly, time pressure does not appear to elevate the priority of later information but may alter the observable influence of temporally ordered information by consolidating the influence of early-presented cues. Nevertheless, this interpretation remains inferential and should be examined in future work using process-level measures. It should be noted that spatial position was randomized and counterbalanced across trials and did not reliably interact with the focal three-way pattern. This design was implemented to control for spatial confounds and to prioritize the examination of time pressure and presentation order. Thus, the key finding in Experiment 2 was that the influence of presentation order on risky choice varied as a function of both time pressure and framing. Overall, Experiment 2 suggests that time pressure plays a moderating role in risky decision-making. Beyond compressing processing depth, time pressure may alter how decision-makers rely on information presentation order, potentially by increasing reliance on early-presented information. When combined with loss framing, this mechanism could substantially amplify the influence of presentation order on risk preference. These results extend those of Experiment 1 by suggesting that presentation order may function as a contextual factor shaping framing effects under time pressure.

## 4. General Discussion

### 4.1. The Effect of Time Pressure on the Framing Effect in Risky Decision-Making

The findings of Study 1 suggest that the magnitude of the framing effect was greater under time pressure than under no time pressure. Participants showed stronger risk aversion in the gain frame and stronger risk seeking in the loss frame when decisions were made under temporal constraints. This pattern is broadly consistent with prior research reporting that time pressure can accentuate framing-related choice tendencies (Olschewski & Rieskamp, 2021; Roberts et al., 2022). Eye-tracking evidence from Study 1 further showed that time pressure was associated with reduced overall information search and altered attentional allocation across processing stages. Specifically, during the early attentional stage, attention allocation exhibited some frame-specific characteristics; in later processing stages, time pressure generally reduced overall information search and appeared to be more closely aligned with information cues that were consistent with the gist of the current decision frame. Importantly, time pressure did not simply increase attention to a particular option; rather, this pattern of attention appeared to align more closely with the frame-diagnostic information. Reaction time results converged with this pattern that decision latencies were substantially longer in the absence of time pressure, particularly in the loss frame. Together, these behavioral and attentional findings indicate that time pressure was associated with both compressed decision latency and altered information acquisition patterns. These findings are consistent with theoretical perspectives emphasizing the role of processing constraints in shaping risky choice. Cognitive Resource Theory (Wedel et al., 2022; Zhou et al., 2024) proposes that temporal constraints may reduce available cognitive resources and increase cognitive load, thereby constraining systematic, verbatim comparisons. Complementing this, Fuzzy-Trace Theory (Reyna et al., 2018) highlights that decision-makers often rely on gist-based representations when making judgments, which is conceptualized as a developmentally advanced and efficient mode of reasoning. Within this framework, limited processing time may prompt reliance on frame-consistent gist representations, thereby potentially contributing to stronger frame-consistent choice patterns under time pressure. Overall, these results suggest that time pressure was associated with patterns that are compatible with greater reliance on gist-based processing. Rather than establishing a specific cognitive mechanism, the present findings offer a theoretically coherent interpretation consistent with existing accounts of constrained processing and heuristic reliance under time limitations.

### 4.2. The Effect of Spatial Position on the Framing Effect Under Time Pressure

Study 1 further examined whether spatial position modulated the framing effect under different time conditions. Under time pressure, the influence of spatial position appeared to be limited. Time pressure was associated with greater attention to frame-relevant information relative to spatial characteristics per se. Consistent with this pattern, spatial layout did not significantly alter the framing effect under temporal constraints. Although prior studies have demonstrated that spatial position can shape visual attention and decision preferences (Ariel et al., 2011; Kwak & Huettel, 2018; Segovia et al., 2025), such positional advantages did not appear to significantly alter the framing effect under the time-constrained conditions of the present study. By contrast, in the absence of time pressure, a significant interaction between spatial position and framing emerged. When the certain option was presented on the left, participants showed a greater tendency to select the risky option, particularly in the loss frame. One possible interpretation is that habitual left-to-right reading and scanning tendencies may increase the likelihood that left-positioned options are attended to earlier in the decision process. Under loss framing, a left-positioned certain option represents a sure loss, which may render the right-positioned risky option more attractive in comparative evaluation. Taken together, these findings suggest that the effect of spatial position on framing is contingent on available cognitive resources. When temporal constraints are relaxed, spatial layout may shape decision outcomes in conjunction with framing cues. Under time pressure, however, the observable influence of spatial position was reduced, indicating that the impact of contextual features can vary across processing conditions.

### 4.3. The Effect of Presentation Order on the Framing Effect Under Time Pressure

To further examine the role of visual presentation factors in risky decision-making, Study 2 manipulated the sequential order in which options were presented. A significant three-way interaction was observed among time pressure, framing, and presentation order. Follow-up analyses revealed that the influence of presentation order on risk preference was condition-specific, emerging only under time pressure and being most pronounced in the loss frame. Specifically, when the certain option was presented second (i.e., the risky option first), participants exhibited greater risk-seeking behavior in the loss frame. This pattern was not observed in the gain frame or under no-time-pressure conditions. Conceptually, this finding appears related to the previously observed position effects. It suggests that information encountered earlier in the decision sequence is more likely to serve as a reference point, thereby influencing how individuals subsequently evaluate whether the option elicits attraction or avoidance (Kwak & Huettel, 2018; Stewart et al., 2016). Under time pressure, processing time may increase reliance on initially presented information, as opportunities for extended comparison are reduced (Kahneman, 1973; Schiebener & Brand, 2015). Particularly in the loss frame, early exposure to the risky option may render it comparatively salient during evaluation within a loss context, which could contribute to heightened risk-seeking tendencies under temporal constraints (Reyna et al., 2018). Importantly, this interpretation is consistent with accounts emphasizing the greater impact of early information when processing resources are constrained rather than enhanced prioritization of later-presented options. Overall, the results suggest that time pressure may interact with the sequential structure of information presentation, such that order effects become more pronounced in specific framing contexts. The early anchoring of initially encountered options under resource-limited conditions may further highlight that the influence of presentation format on risky choice depends on both framing and processing conditions.

## 5. Limitations and Outlook

Although this study has provided some new insights for the existing literature, several limitations should be acknowledged.

First, while the experimental task ensured precise control, it was hypothetical and constrained to laboratory conditions. Moreover, the time-pressure manipulation involved differences in trial-level feedback frequency, which may have engaged additional motivational processes. The fully randomized spatial layout adopted to control positional confounds may have attenuated the detectability of stable position effects. Future research could address these design trade-offs by orthogonally manipulating time constraints and feedback structure in real-incentive paradigms and by implementing systematic spatial arrangements to more directly examine how spatial attention interacts with temporal constraint.

Second, although eye-tracking data provided evidence regarding attentional allocation, the proposed mechanisms, such as increased reliance on early information under time pressure, were not directly tested. The interpretations offered remain theoretically informed but indirect, and future studies employing process-tracing methods, computational modeling, or experimental manipulations specifically targeting reference formation and comparison dynamics would allow for a more direct examination of the mechanisms involved.

Third, the present study also conducted exploratory analyses of higher-order interactions, including the four-way interaction among time pressure, framing, spatial position, and presentation order. These analyses provide preliminary evidence regarding the complexity of contextual influences on risky choice and highlight promising directions for future research. Subsequent studies could adopt confirmatory factorial designs and independent replication to further evaluate the robustness of these interaction patterns.

Finally, the sample consisted primarily of college students, which limits the generalizability of the findings. Future research should include participants from broader age, cultural, and occupational backgrounds to examine how individual differences, such as cognitive style or risk attitude, moderate sensitivity to framing effects.

## 6. Conclusions

This study integrates behavioral and eye-tracking evidence to examine how time pressure shapes framing effects in risky decision-making. The findings indicate that time pressure is associated with constrained information search and stronger frame-consistent choice patterns. Patterns of visual attention allocation were closely linked to these behavioral differences, suggesting that attentional distribution may play an important role in how framing effects are expressed under temporal constraints. In addition, visual presentation factors interacted with time pressure and framing, highlighting the context-dependent nature of risky choice. Together, these results contribute to a more nuanced understanding of how temporal constraints and presentation structure influence decision processes in framed risk situations.

## Figures and Tables

**Table 1:** Repeated-measures ANOVA on the proportion of risky choices.

Analysis	Effect	*F*(1, 25)	*p*	$\eta_{p}^{2}$
Time Pressure × Frame	Time Pressure	7.55	<0.05	0.23
	Frame	282.58	<0.001	0.92
	Time Pressure × Frame	309.50	<0.001	0.93
Time Pressure × Frame × Position	Time Pressure	0.47	0.501	0.02
	Frame	117.92	<0.001	0.83
	Position	6.32	<0.05	0.20
	Time Pressure × Frame	64.72	<0.001	0.72
	Time Pressure × Position	2.62	0.118	0.09
	Frame × Position	3.95	0.058	0.14
	Time Pressure × Frame × Position	0.75	0.394	0.03
Position × Frame (No Time Pressure)	Position	9.74	<0.01	0.28
	Frame	35.63	<0.001	0.59
	Position × Frame	6.07	<0.05	0.20

**Table 2:** Repeated-measures ANOVA analysis of the effects of time pressure, position, and presentation order on the framing effect.

	*F*(1, 23)	*p*	$\eta_{p}^{2}$
Time Pressure	0.87	0.36	0.04
Frame	32.72	<0.001	0.59
Presentation Order	8.02	<0.01	0.26
Position	0.73	0.40	0.03
Time Pressure × Frame	2.96	0.10	0.11
Time Pressure × Presentation Order	27.85	<0.001	0.55
Frame × Presentation Order	0.30	0.59	0.01
Time Pressure × Position	2.06	0.17	0.08
Frame × Position	0.02	0.88	<0.001
Presentation Order × Position	4.95	<0.05	0.18
Time Pressure × Frame × Presentation Order	13.58	<0.01	0.37
Time Pressure × Frame × Position	0.44	0.51	0.02
Time Pressure × Presentation Order × Position	0.22	0.64	0.01
Frame × Presentation Order × Position	0.96	0.34	0.04
Time Pressure × Frame × Presentation Order × Position	0.01	0.94	<0.001

## Data Availability

The raw data supporting the conclusions of this article will be made available by the authors on request.
